# White lie during patient care: a qualitative study of nurses’ perspectives

**DOI:** 10.1186/s12910-020-00528-9

**Published:** 2020-09-03

**Authors:** A. Nikbakht Nasrabadi, S. Joolaee, E. Navab, M. Esmaeili, M. Shali

**Affiliations:** 1 School of Nursing and Midwifery, Tehran University of Medical Sciences, Nosrat St., Tohid Sq, Tehran, 141973317 Iran; 2 Nursing Care Research Center, Iran University of Medical Sciences, Tehran, Iran; 3 Center for Evaluation & Outcome Sciences (CHEOS), University of British Columbia, Vancouver, Canada

**Keywords:** Ethics, White lie, Truth-telling, Nurse, Content analysis

## Abstract

**Background:**

Keeping the patients well and fully informed about diagnosis, prognosis, and treatments is one of the patient’s rights in any healthcare system. Although all healthcare providers have the same viewpoint about rendering the truth in treatment process, sometimes the truth is not told to the patients; that is why the healthcare staff tell “white lie” instead. This study aimed to explore the nurses’ experience of white lies during patient care.

**Methods:**

This qualitative study was conducted from June to December 2018. Eighteen hospital nurses were recruited with maximum variation from ten state-run educational hospitals affiliated to Tehran University of Medical Sciences. Purposeful sampling was used and data were collected by semi-structured interviews that were continued until data saturation. Data were classified and analyzed by content analysis approach.

**Results:**

The data analysis in this study resulted in four main categories and 11 subcategories. The main categories included hope crisis, bad news, cultural diversity, and nurses’ limited professional competences.

**Conclusion:**

Results of the present study showed that, white lie told by nurses during patient care may be due to a wide range of patient, nurse and/or organizational related factors. Communication was the main factor that influenced information rendering. Nurses’ communication with patients should be based on mutual respect, trust and adequate cultural knowledge, and also nurses should provide precise information to patients, so that they can make accurate decisions regarding their health care.

## Background

In Iran, medical ethics literature and the Patient’s Bill of Rights highlight patients’ right to receive accurate and complete information about diagnosis, prognosis, and treatments [1–4]. Iran is an Islamic country in which people are advised to tell the truth and prohibited from telling lies [5]. Although healthcare providers and patients have the same viewpoint about truth telling in the process of treatment [4], there are sometimes emotional, professional, and/or cultural barriers to the provision of accurate information to patients [5]. In any of aforementioned circumstances, healthcare providers may inevitably tell lies which are called white lies or therapeutic fibs [6].

By definition, a white lie is a deceptive interaction to prevent injury or grief or to protect people’s feelings [7–11]. History of medicine shows ample evidence in which Greek physicians did not reveal the whole information to patients or provided them with inaccurate one to get them to accept treatments [9]. Hippocrates’ notes show that truth-telling or accurate information provision to patients about the outcome of an illness can aggravate prognosis [12].

Although telling a lie is an unethical action, it is not a person-oriented practice and hence, its prevention and management necessitate some interventions to manage its underlying causes [8]. Physicians are the main information source to patients and their families [13–16]. Moreover, truth-telling necessitates all healthcare providers, particularly nurses to be involved in this process [16]. Studies showed that nurses are in a position that have to hide truths frequently [17, 18]. They sometimes are placed in situations where truth-telling was impossible so there is no other way left for them but to tell white lies [19].

In this field, most studies have been conducted with the aim of examining the attitude of target groups towards telling the truth in a form of quantitative or literature review. Yet, no study has been found in Iran to use qualitative methods to examine the experiences and perspectives of care providers in Iranian cultural context. In addition, there is no in-depth information about the situations in which nurses feel compelled to tell a white lie. The present study was conducted to address this gap and aimed to explore nurses’ experiences of telling a white lie during patient care.

## Methods

### Design

This qualitative descriptive study was conducted from June to December 2018 using conventional content analysis approach. Qualitative content analysis is a suitable method when the purpose of a study is to extract the content of a text, as it facilitates the identification and categorization of the information without changing its meaning [20].

### Sample and setting

Study participants were nurses who were working in ten state-run educational hospitals affiliated to Tehran University of Medical Sciences, Tehran, Iran. These hospitals have the highest rate of patient admission with different diagnoses. Sampling was purposively done with maximum variation in terms of participants’ gender, educational level, work experience, and work environment. Inclusion criteria were associate degree or higher in nursing, agreement for participation in the study, ability to communicate in Persian language and share personal experiences.

### Data collection

Data were collected by the first author (of this paper) through in-depth individual semi-structured interviews. Relevant field notes were written before and after interviews by the interviewer and during following interviews for clarification. Sample interview questions include; “Have you ever experienced a situation during patient care where you did not want or could not tell the truth to your patients?” “Would you please explain it?” “Which tricks did you use in such situations?” “In what situations during patient care did you tell a white lie?” “Would you please explain your experiences of telling a white lie during patient care?” “How do you describe telling a white lie during patient care?”. Interviews were held at participants’ preferred time and place and lasted between 30 and 60 min. Data collection continued until reaching data saturation after the sixteenth interviews. Two more interviews were also conducted to ensure the data saturation. Interviews were digitally recorded with voice recorder (Sony- ICD-UX560F) and transcribed verbatim by the corresponding author.

### Data analysis

Data were analyzed through five-step conventional content analysis method proposed by Graneheim and Lundman [20]. In the first step, each interview was transcribed word by word. In the second step, the interview transcript reviewed several times to obtain a sense of the whole. In the third step, each interview transcript was considered as the unit of analysis, then meaning units were identified and coded. The first author analyzed the total data, while the second one analyzed half of the textual data. Two authors then compared the codes, and revised minor disagreements after discussion. In the fourth step, codes grouped into subcategories according to their conceptual similarities and differences.

In the fifth step, subcategories compared with each other and the latent data content identified and presented as the main categories. The final four categories were examined by all authors to ensure a clear difference between categories and subcategories and fit the data within each category.

Parts of the audiotape were translated from Farsi into English by an independent translator blind to the study to check for consistent translation. Data analysis carried out using MAXQDA statistical software version 2010.

### Trustworthiness

Trustworthiness was applied with Guba and Lincoln criteria of credibility, dependability, confirmability, and transferability [21]. Credibility was established using member- and peer-checking, prolonged engagement, and maximum variance of participants’ selection. For instance, for member-checking, a brief report of the findings was given to two clinical nurses, whom they were asked to reflect their experiences and perspectives to the analysis report for researcher assurance. For peer-checking, two qualitative researchers approved the primary codes and categorizing process. Transferability achieved via the provision of a rich description of data collection, analysis processes and findings to allow the readers to match the findings with their contexts.

## Results

Participants were 12 female and six male nurses with the mean age of 37 ± 4.2 years old and the mean work experience of 13 ± 4.6 years. Totally, in data analysis, 314 codes were generated which further categorized into four following main categories and 11 subcategories. The mail categories were the crisis of hope, bad news, cultural diversity, and nurses limited professional competence. These categories are presented in Table 1 and are explained as follows:

**Table 1:** Situations of using a white lie during patients care

Subcategories	Categories
Loss of beliefs	The crisis of hope
Lack of motivation for treatments	
Death anxiety	
News about the diagnosis of a serious illness	Bad news
News about treatment ineffectiveness	
News about significant losses	
Patient’s culture	Cultural diversity
Organizational culture	
Limited communication skills	Nurses’ limited professional competence
Limited professional knowledge	
Limited professional experience	

### The crisis of hope

Hope is an antidote that makes illnesses and their difficulties bearable. Our participants took part in situations where their clients experienced the crisis of hope after hearing about truths related to their illnesses. Therefore, they felt compelled telling white lies. This category subcategorized into three: loss of beliefs, lack of motivation for treatments, and death anxiety.

#### Loss of beliefs

Patients’ beliefs may change during illness. Awareness of bitter truths may challenge or change their beliefs. Beliefs, in turn, affect patients’ perceptions of health and illness. According to the participants, a white lie helps nurses reduce the importance of negative situations and supports patients’ beliefs.

*When we inform them about the bitter truths, they lose their faith in treatments, dietary regimen, and even religion and God (P. 14).*

#### Lack of motivation for treatments

In case of serious illness or lifetime treatment, motivation is a key factor affecting treatment success and patient adherence to treatments. Our participants referred to tell a white lie or avoid truth-telling as strategies for maintaining patients’ motivation.

*A question which patients always ask is, “Will I recover from this disease?” The answer is sometimes “No”. But who can give this answer forthrightly? It will be associated with motivation loss. Thus, we need to use answers like, “Go ahead; it may get better. The science is advancing” (P. 11).*

#### Death anxiety

Awareness of imminent death can cause an acute psychological crisis for patients and reduce their collaboration and motivation. Moreover, death anxiety can negatively affect hope and quality of life. All these situations may require healthcare providers to tell a white lie.

*Family members may warn us about the fact that their patient fears cancer and ask us not to tell him/her the truth. Thus, we should use other words in these cases to prevent patient anxiety or fear over death from affecting his/her hope. For instance, we may use words such as gastric ulcer or tumor instead of the word cancer (P. 13).*

### Bad news

One of the most challenging situations of telling a white lie is when nurses want to give patients and family members bad news. In these situations, nurses may choose to tell a white lie due to their lack of knowledge about strategies for giving bad news, concern over damages to nurse-patient relationships, unfamiliarity with patients’ morale and emotions, and fear over patients’ strong emotional reactions. Situations in which nurses preferred to tell a white lie for giving bad news were related to the diagnosis of a serious illness, treatment ineffectiveness, and significant losses.

#### News about the diagnosis of a serious illness

Getting informed about diagnoses that are publicly equated with an imminent death makes these difficult situations even more challenging and may shock patients and families. In these situations, nurses may tell a white lie to minimize the effects of the shock associated with hearing about a piece of bad news.

*Particularly, in the case of the diagnosis of cancer, multiple sclerosis, and similar serious illnesses, we need to play with words to avoid telling the truth about the diagnosis (P. 9).*

#### News about treatment ineffectiveness

Long-term chemotherapy courses, major surgeries, and extensive treatments may cause patients to perceive that they are approaching recovery. However, when treatments are ineffective, nurses face challenges and difficulties in telling patients about treatment ineffectiveness and may resort to white-lie-telling.

*When futile treatments are continued, patients may conclude that they are achieving recovery. They may ask us about treatment effectiveness. At that moment, we cannot tell them about treatment failure (P. 10).*

#### News about significant losses

Significant losses such as loss of a child, an organ, or a family member are very stressful for patients and their family members. Nurses who break the news about significant losses to patients and family members may face unexpected emotions such as shock, anger, belief loss, deep grief, and guilt. Accordingly, they may primarily tell a white lie to reduce such emotions.

*When a patient dies and we want to inform his/her family members over the phone, we cannot directly tell them that the patient has died; rather, we just tell them that the patient is not in good condition and ask them to quickly refer to the hospital (P. 2).*

### Cultural diversity

People with different cultures and ethnicities have different methods for disclosing information about illness-related realities and have different rituals for dealing with reality. Besides culture and ethnicity, each person has a unique method for dealing with reality. The two subcategories of the cultural diversity main category are the patient’s culture and organizational culture.

#### Patient’s culture

Nurses need to provide care to patients from different cultures. Because of their cultural beliefs, patients have their unique behaviors, some of which may not be in line with treatment goals. Thus, nurses may sometimes feel compelled to tell a white lie to achieve the treatment goals.

*There was a child in our ward with a nasogastric tube in place and a “Nothing by mouth” order. His family members brought us an admixture from their home city and believed that the admixture could treat their child. They firmly insisted on the gavage of the admixture while the child should not receive anything by mouth due to his medical conditions. Finally, we had no option but to tell the family that we had given the food to their child (P. 16).*

#### Organizational culture

Moreover, organizational culture, values, and beliefs affect their behaviors. According to our participants, organizational culture and policies may require them to tell a white lie.

*Even in case of the diagnosis of serious illnesses, we are not allowed to tell the families anything until the physicians inform them. In those situations, we answer patients’ questions without referring to reality (P. 18).*

### Nurses’ limited professional competence

In addition to the characteristics of patients, healthcare organizations, and other healthcare providers, nurses’ limited professional competence also affected their use of white-lie-telling. This main category included three subcategories, namely limited communication skills, limited professional knowledge, and limited professional experience.

#### Limited communication skills

Communication is the core of nursing care. In difficult situations when nurses are the only accessible source of information for patients, limited communication skills may require them to tell a white lie.

*Sometimes, patients ask questions that I don’t know how to answer. In these situations, I attempt to provide good answers; however, occasionally I cannot manage the situation and cannot tell the truth without annoying the patient. Thus, I may feel compelled to use a white lie (P. 12).*

#### Limited professional knowledge

Medical and nursing sciences continuously advance and change. Sometimes, nurses do not have adequate knowledge about patients and their treatments and hence, may find themselves in situations that require them to use a white lie.

*Sometimes, I may not know the answers to patients’ questions. In such situations, I may have no option but to use a white lie. Of course, this is not true for critical situations (P. 15).*

#### Limited professional experience

Experience helps nurses understand which information should be given to patients and which strategies should be used for giving information. Novice nurses are more prone to situations that force them to tell a white lie.

*More experienced nurses have magic sentences which are neither a lie nor direct answers to patients’ questions. At the beginning of my work, I didn’t have experience and told the truth to the patients directly. Such direct truth-telling caused negative consequences. After a while, I sometimes felt compelled to use a white lie to answer some patients’ questions (P. 6).*

## Discussion

This study explored nurses’ experiences of the situations of telling white lie during patient care. Our findings showed that nurses might feel compelled to use a white lie during patient care due to factors such as the crisis of hope, bad news, cultural diversity, and nurses’ limited professional competence.

Hope crisis is one of the main categories of this study. Although nurses experienced fear of hope crisis in case when they were obliged to tell a bitter truth, another study by Seyedrasooly et al. has shown neutral effects on patients’ hope and quality of life of the patient in truth disclosure situations [22]. In Apatira et al. [23] study, overall, 93% (166 of 179) of surrogates felt that avoiding discussions about prognosis is an unacceptable way to reduce death anxiety and maintain patients’ hope. In another study, awareness of bad news had no effect on treatment motivation or patients’ beliefs. Patients and their families were concerned about how to report bad news [24] To overcome this concern, techniques such as information provision about available diagnostic procedure and treatments, supporting systems and allocating enough time based on each patient’s personal needs should be taken [25].

Bad news is the second main category of telling a white lie during patient care to reduce or avoid patients’ reactions. Patient reactions to bad news are not predictable and may include anger, crying, denial, verbal abuse, threatening behaviors, bargaining, and silence. Management of all these reactions requires great communication skills [26]. Bagherian et al. showed that nurses were reluctant to tell bitter truths to patients and did not have the necessary abilities to do so [27]. Also, Gauthier et al. showed further reasons for white lie use in bad news break like caregivers’ negative feelings, time management, accurate information provision, and ability to provide logical answers to patients’ and their families’ questions by the nurses [28].

Culture is another factor that force the nurses to use white lie. In this study culture has been reflected in different areas of medical, nursing, organizational and patient personal aspects. White lies have been used to achieve therapeutic goals where abovementioned cultural aspects stood as an obstacle against these goals. However, based on Iranian and Islamic culture, telling the truth is a religious virtue and strongly recommended. This principle signifies the importance of patients dignity and latitude [29], and highlights the nursing supporting role against bad news harms. Despite this religious and national virtue, nurses’ experiences showed that cultural limitations and differences made truth-telling an intricate task especially in patient critical situations. Truth-telling to patients seems to be easy in countries such as the United States [30]. However, that would be difficult and requires more advanced communication skills in Asian and southeastern European countries. So, given the impact of culture on the acceptance of truth, medical and nursing educational authorities need to develop strategies to improve nurses’ competence in truth-telling and patient informational provision.

In addition to organizational and patient-related factors, nurses’ limited professional competence also contributed to their use of white lie during patient care. Our findings also showed nurses related factors for white lie use such as limited communication skills, professional knowledge, and professional experience. The ability to understand patients’ wordings and their insights about treatment modalities together with appropriate physical, emotional, and social environment all can help to establish rational and effective communication techniques and facilitate truth-telling to patients and their families [31].

In all, dilemma between telling the truth or white lie is an ethical challenge that cannot be overcome only with improved personal ability. To reach this goal, organization supportive atmosphere may drive nurses to cope with ethical challenges of white lie in the patient care setting. Malloy et al. suggest that nurses’ work environment can affect their attitudes toward ethical issues and their moral decision-making [32]. Organizational support and nurse leaders’ supportive behavior play key roles in nurses’ productivity and their ethical performance promotion [33].

### Limitations

One of the limitations of this study was the limited number of hospitals, which were affiliated hospitals of Tehran University of Medical Sciences. Further studies in other therapeutic settings can provide valuable data from other nurses which will lead to more generalized findings. This study also only addressed the perspectives and experiences of nurses, while the patients’ own experiences and perspectives were not investigated, which limited the more comprehensive examination of the white lies in the patient care process. Therefore, it is recommended to consider patients’ opinions and experiences in future studies.

## Conclusion

This study suggests that a wide range of patient-oriented, nurse-related, and organizational factors may require nurses to tell a white lie during patient care. Nurses need to develop their communication skills and experiences to establish effective communication with patients and their families to provide them with accurate information. Communication needs to be established based on adequate patients’ cultural knowledge and organization supportive actions. Our findings highlighted the importance of truth-telling and effective communication skills to reduce white lie for information provision in different medical setting especially in dilemmatic situations. This study can be used as the basis for further quantitative and qualitative studies of telling white lie and its consequences in patient care setting.

## Supplementary information

**Additional file 1.** Interview questions

## Data Availability

Data are available by contacting the corresponding author.
