# Supplementary material for: White lie during patient care: a qualitative study of nurses’ perspectives
Source: BMC Med Ethics. 2020 Sep 3;21:86. doi: 10.1186/s12910-020-00528-9 (PMC7470607; doi:10.1186/s12910-020-00528-9)
Supplement: Supplementary file 1 — Additional file 1. Interview questions [file 12910_2020_528_MOESM1_ESM.docx]

**Interview questions**

“Have you ever experienced a situation during patient care where you did not want or could not tell the truth to your patients?”

“Which tricks did you use in such situations?”

“In what situations during patient care did you use a white lie?”

“Would you please explain about your experiences of telling a white lie during patient care?”

“How do you define telling a white lie during patient care?”.
